# Prevalence and Correlates of Motoric Cognitive Risk Syndrome in Chinese Community-Dwelling Older Adults

**DOI:** 10.3389/fragi.2022.895138

**Published:** 2022-06-30

**Authors:** Anying Bai, Weihao Xu, Zhanyi Lin

**Affiliations:** ^1^ Department of Epidemiology and Biostatistics, School of Population Medicine and Public Health, Chinese Academy of Medical Sciences and Peking Union Medical College, Beijing, China; ^2^ Department of Cardiology, Guangdong Provincial Cardiovascular Institute, Guangdong Provincial People’s Hospital, Guangdong Academy of Medical Sciences, Guangzhou, China; ^3^ Department of Geriatrics, Guangdong Provincial Geriatrics Institute, Guangdong Provincial People’s Hospital, Guangdong Academy of Medical Sciences, Guangzhou, China

**Keywords:** prevalence, motoric cognitive risk syndrome, associated factors, community-dwelling elderly, China

## Abstract

**Background:** Motoric cognitive risk (MCR) syndrome is considered to be a pre-dementia syndrome. Although an increasing number of studies have begun to focus on this syndrome, few investigations have been launched in China. This study was performed to examine the prevalence and correlates of MCR in China.

**Methods:** We included 5,725 adults aged over 60 years from China Health and Retirement Longitudinal Study (CHARLS). MCR was defined as the presence of subjective cognitive complaints and a gait speed ≤20th percentile of the weighted population distribution adjusted for sex and height. The associations among selected modifiable associated factors and clinical measures with MCR were examined using multivariate logistic regression analysis.

**Results:** Of the participants, 414 met the criteria for MCR with an overall prevalence 7.29% (95% CI: 6.62–7.96%). MCR was found to be more prevalent among women than men (9.73 vs 4.85%), and more prevalent among participants ≥75 years than those <75 years (7.85 vs 5.23%). After multivariable adjustment, lower or upper extremity functional limitations, activities of daily living (ADL) disabilities, weak grip strength, exhaustion, and history of hypertension were found to be significantly associated with MCR. The multivariate analysis also showed higher levels of cystatin C and C-reactive protein were associated with increased odds for MCR.

**Conclusions:** The present study showed that MCR syndrome is highly prevalent among Chinese community-dwelling older adults, and revealed several factors that were correlated with MCR. Longitudinal studies are warranted to further explore the modifiable risk factors of MCR.

## Introduction

Motoric cognitive risk (MCR) syndrome is featured by subjective cognitive complaints and slow gait among older individuals (Verghese et al., 2019) and is considered to be a pre-dementia syndrome resemble mild cognitive impairment (MCI). However, while the diagnosis of MCI requires a time-consuming comprehensive neuropsychological assessment, diagnosis of MCR does not. This permits easier detection of members of the older population who are at risk for dementia. Previous studies have associated MCR syndrome with a wide range of adverse events, including dementia (Chhetri et al., 2017), frailty (Sathyan et al., 2019), falls (Callisaya et al., 2016), disability (Doi et al., 2017), and all-cause mortality (Ayers and Verghese, 2016; Beauchet et al., 2019; Maggio and Lauretani, 2019). Considering that effective treatment methods for dementia are still lacking, it is critically important to focus on pre-dementia syndromes and their modifiable risk factors to identify opportunities for early interventions to reduce the incidence of dementia.

China is the world’s most populous country. It is estimated that the number of Chinese people aged 80 years or older will quadruple over the next 35 years from 22.6 million in 2013 to 90.4 million in 2050 (Hu et al., 2018; Jia et al., 2020). The prevalence of dementia will increase in parallel to the population age. Dementia imposes heavy burdens on families, the healthcare system, and society (Jia et al., 2020). Several studies in different countries have reported the prevalence of and risk factors associated with MCR syndrome. An MCR prevalence study conducted across 17 countries reported a pooled prevalence of 9.7% globally (Verghese et al., 2014), and a cross-sectional study among Japanese older adults reported a prevalence of 6.4% (Doi et al., 2015). To date, only two studies have researched the prevalence and risk factors associated with MCR in China, examining a province-level and a city-level cohort, respectively. A full-scale picture of the epidemiology of MCR syndrome in China would help identify subgroups of older Chinese adults among whom MCR syndrome occurs more frequently than in the general population, thereby enhancing our understanding of potential risk factors for MCR syndrome among the Chinese elderly population.

In the present study, China Health and Retirement Longitudinal Study (CHARLS), a nationally representative prospective study of the community-dwelling Chinese population aged 45 years and over was used to determine the prevalence of MCR in China and to examine associated factors, such as lifestyle factors, physical function, and biomarker correlates, of MCR.

## Methods

### Participants

**FIGURE 1:**
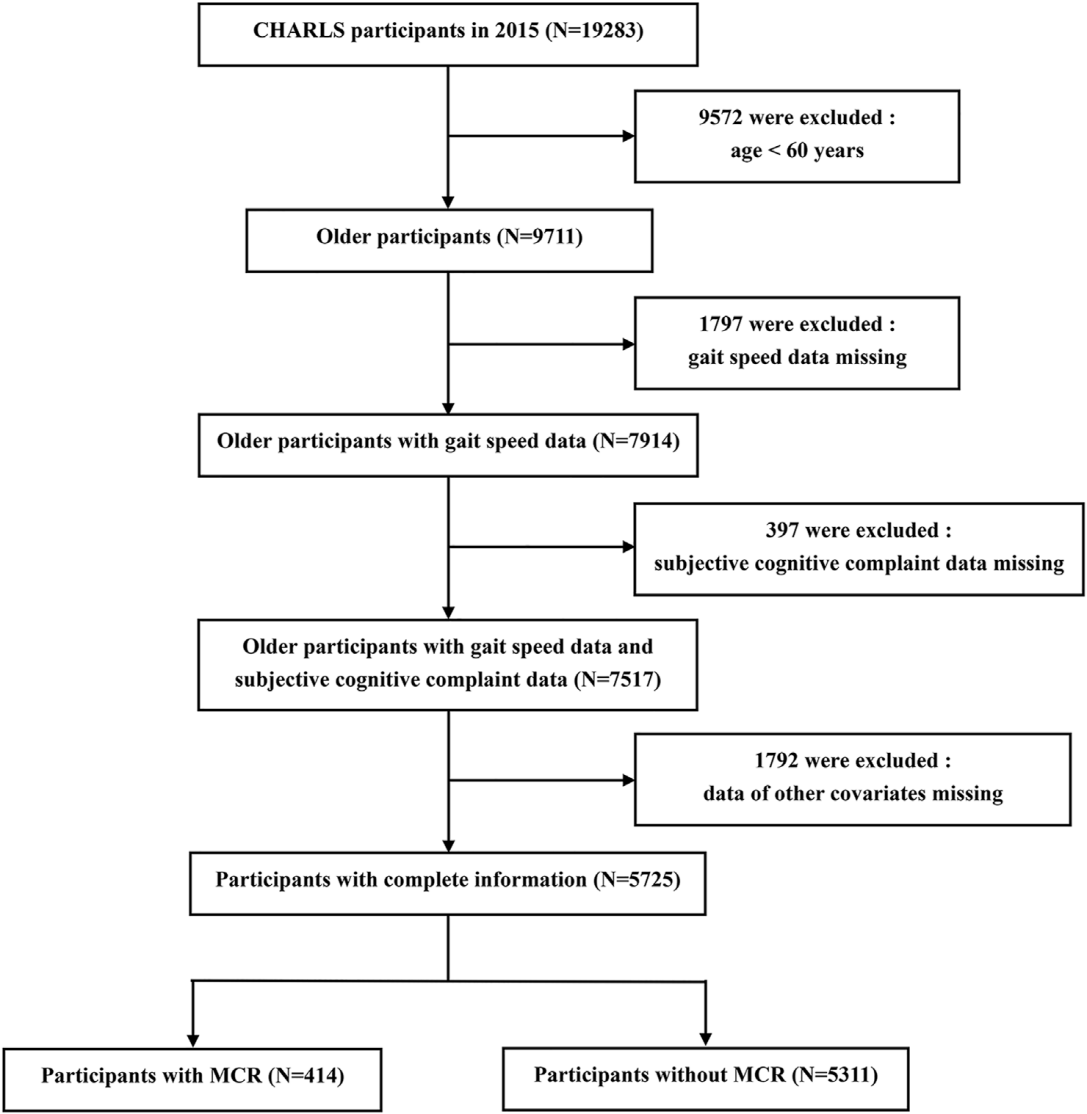
Inclusion process of study population.

Subjects were participants of CHARLS, an ongoing longitudinal cohort study of a nationally representative sample of community-dwelling adults (age ≥45 years) from 28 provinces in China. CHARLS collected information including basic demographics, family information, health status, health care, employment and household economy. A total of 17,705 respondents were interviewed at the baseline survey in 2011, and they were followed every 2 years using a face-to-face, computer-assisted personal interview (CAPI) technique. Additional details about the recruitment strategy, design, and sampling approaches of the CHARLS have been previously documented (Zhao et al., 2014). A total of 19,283 respondents were interviewed in 2015, and 9,572 of them were excluded due to age <60 years. We further excluded those who did not have complete data on gait speed (N = 1797), subjective cognitive complaint (N = 397) or other covariates (N = 1792). Therefore, the final sample included in the prospective analysis contained 5,725 participants. More details of the inclusion process of the study population are provided in Figure 1. The original CHARLS was approved by the Ethical Review Committee of Peking University (IRB00001052–11015), and all participants signed the informed consent at the time of participation.

### Definition of Motoric Cognitive Risk Syndrome

The diagnosis of MCR syndrome was founded on the original criteria proposed by Verghese and others (Zhang et al., 2020), defined as cognitive complaints and slow gait speed without dementia or impaired mobility. In our study, cognitive complaints were assessed using a self-reported question about memory loss: “How would you rate your memory at the present time?” Respondents were asked to rank their answer on a scale of five values: excellent, very good, good, fair, and poor. Those who reported fair or poor were recorded as having cognitive complaints. The average time respondents took to walk along a straight path two times was used to compute gait speed. Usual gait speed on a 2.5-m course was measured objectively, and was used to assess physical performance. We defined slow gait speed as being ≤ the 20th percentile of the weighted population distribution, adjusted for sex and height.

### Modifiable Associated Factors and Covariates

Based on previous studies of MCR and dementia risk factors in China, we selected and examined several possible factors associated with MCR, including lifestyle factors, physical activity, disability, self-reported functional limitations, components of frailty, physical performance, medical conditions, and clinical measures.

### Lifestyle Factors

In this study, the following lifestyle factors were selected and examined based on previous studies that have linked them to either MCR or dementia risk: body mass index (BMI: kg/m^2^), obesity, smoking, and alcohol consumption. Height and weight were measured and used to calculate BMI using the standard formula. Overweight was defined as a BMI value of 25 or greater, as previously defined among the Chinese elderly population (Pi-Sunyer et al., 1998). All participants were classified into two groups: no drinking/smoking or former drinking/smoking and current drinker/smoker.

### Physical Activity

Physical activity (PA) was assessed by analyzing the amount of time a person engaged in different types of physical activity (vigorous activity, moderate activity, and walking for at least 10 min continuously) in a usual week. The PA scores were constructed by multiplying the number of days and the daily PA duration index for each activity, based on the methodology employed in the International Physical Activity Questionnaire (IPAQ) (Craig et al., 2003). Participants met criteria for physical inactivity if they self-reported that they did not engage in vigorous, moderate, low levels of activity or walk 10 or more minutes continuously during a usual week.

### Disability

Five activities of daily living (ADL) tasks (dressing, bathing, eating, getting out of bed, and going to the toilet) were used to assess disability. For each ADL task, participants were asked, “Do you have difficulty in performing the task?” Those participants who responded, “I have difficulty but can still do it,” “Yes, I have difficulty and need help,” or “I cannot do it” to one or more of the ADL tasks were supposed to have an ADL disability.

### Self-Reported Functional Limitations

Self-reported functional limitations were also measured in this study. Participants were considered to have lower extremity functional limitations if they had difficulty performing any of the following tasks on a regular basis: getting up from a chair after sitting for a long period, climbing several flights of stairs without resting, stooping, kneeling, or crouching. Participants were classified as having upper extremity functional limitations if they reported difficulty in any of the following tasks: reaching or extending arms above the shoulder level, lifting or carrying weight exceeding 5 kg, or picking up a small coin from a table. For each task, participants were asked, “Do you have difficulty in performing the task?” Participants who responded, “I have difficulty but can still do it,“, “Yes, I have difficulty and need help,” or “I cannot do it” were supposed to have difficulty.

### Components of Frailty

Frailty was first described by Fried and others (Fried et al., 2001) in terms of its physical characteristics, or ‘phenotype’, and is objectively identified as three or more of five components: weakness (low grip strength), slowness (slow walking speed), shrinking (unintentional weight loss), exhaustion (self-reported), and low physical activity. Participants were considered as having exhaustion if they answered, “A moderate amount of time; 3,4 days” or “Most of the time” to either of the following two questions from the modified Center for Epidemiological Studies- Depression (CES-D) scale (13): “I could not get going” and “I felt everything I did took great effort.” Other components of frailty included weakness and inactivity were replaced by similar covariates like physical performance (grip strength) and physical activity in our study. Hence, we did not assess frailty as a single measure. Slow gait speed (slowness, one of the five components of frailty) was the component of MCR diagnosis.

### Physical Performance

In this study, physical performance was assessed by handgrip strength and five times sit-to-stand (FTSS) test times by a trained evaluator. The participants were asked to use their dominant hand twice with maximum effort, and the mean of the two test values was used for analysis. The FTSS test procedure was carried out following standardized verbal instructions.

### Medical Conditions

Medical conditions, including history of hypertension, diabetes, and cardiac disease, were all self-reported.

### Clinical Measures

Clinical measures were also used in this study. We used an automatic BP monitor to measure blood pressure (BP) while respondents were in a seated position. Three measurements were taken 45s apart from each other, and the average of the three values was used for analysis. Fasting blood samples were collected by trained nurses in township hospitals or local offices of the China Center for Disease Prevention and Control (CDC). Blood biomarkers included C-reactive protein (CRP; mg/L), blood cell count (10^3^/cm), fasting glucose (mg/dl), platelets (10^3^/cm), hematocrit (%), hemoglobin (g/dl), triglycerides (mg/dl), cystatin C (mg/L), low-density lipoprotein cholesterol (LDL cholesterol; mg/dL), high-density lipoprotein cholesterol (HDL cholesterol; mg/dL) and total cholesterol (mg/dl).

### Cognitive Function

CHARLS included items for cognitive function similar to those used in the Health and Retirement Study, which were components of the Telephone Interview of Cognitive Status battery (Crimmins et al., 2011). The Telephone Interview of Cognitive Status is a telephone version of the Mini-Mental State Examination and assesses cognitive function, and the analysis by McArdle et al. (McArdle et al., 2007). suggested two factors to adequately capture cognitive function: one factor related to episodic memory and a second factor related to other tasks including items on orientation, visuoconstruction, and numeric ability. Similarly, the cognitive dimensions of episodic memory and executive function were measured in this study (Bender et al., 2017). The cognitive dimensions of episodic memory and executive function were assessed by a composite battery of cognitive tests in CHARLS. The global cognition score was the sum of the two test scores and ranged from 0 to 21.

In the episodic memory test, the individuals were asked to recall words immediately (immediate recall) and 5 min later (delayed recall) after interviewers read 10 Chinese nouns to them. The episodic memory score was the average score of the immediate recall and delayed recall tests and could range from 0 to 10. The measure of executive function was based on the Telephone Interview of Cognitive Status (TICS), an 11-item screening test including serial subtractions of 7 from 100 (up to 5 times), date (month, day, and year and season), the day of the week, and the ability to redraw a picture shown to him/her. The total scores representing the number of correct answers could range from 0 to 11.

### Statistical Analysis

Baseline characteristics between “participants with MCR” and “participants without MCR” were compared using unpaired t or χ2 tests. MCR prevalence was compared between sex and age groups using the χ2 test. The independent variables included the selected modifiable associated factors (lifestyle factors, physical activity, disability, self-reported function limitation, components of frailty, physical performance, and medical conditions). Multivariate logistic regression analysis in two models was used to analyze the associations between MCR (dependent variable) and independent variables, and adjusted odds ratios (ORs) and 95% confidence intervals (95% CIs) were reported. We tested each independent variable individually adjusted for demographic data (age, sex, living area, marital status, and education) in baseline Model 1 and included all independent variables and the same set of demographic variables in the fully adjusted Model 2.

We estimated the mean (median if the distribution was highly skewed) level of each biomarker according to health status and used analysis of variance (ANOVA) or nonparametric equivalents to determine whether biomarker levels varied according to MCR syndrome status. We also dichotomized biomarkers using the highest or the lowest quintile (whichever indicated a harmful level) of the sample distribution or clinically relevant cut-points. The χ2 test was utilized to identify whether these proportions varied by MCR syndrome status. Multivariate logistic regression analysis was also performed when univariate analysis showed statistically significant difference (*p* < 0.05) between groups of each biomarker.

All reported *p* values were two-tailed with a significance level of 0.05. All analyses were performed using STATA software (version 14.0; Stata Corp LP. TX).

## Results

**FIGURE 2:**
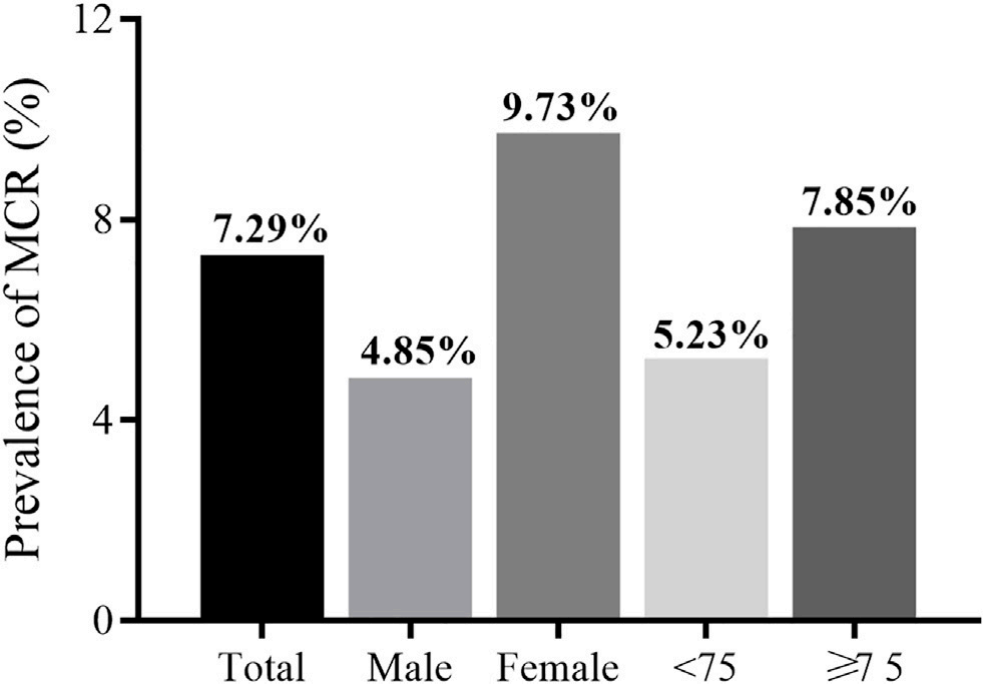
Prevalence of MCR of total study population and different subgroups.

Of the 5,725 subjects (50.1% male, mean age: 67.9 years), the overall prevalence of MCR was 7.29% (95% CI: 6.62–7.96%). There was a lower prevalence of MCR within the male population (4.85%) compared to the female population (9.73%, *p* < 0.001). Participants aged 75 years or older had a higher prevalence of MCR than those aged below 75 years (7.85 vs 5.23%, *p* < 0 0.001). Figure 2 presents MCR prevalence by sex and age. Table 1 presents the characteristics and its comparison between groups. Marital status, BMI, and history of diabetes were not significantly different between groups. All other selected characteristics differed significantly between groups (*p* < 0.05). The characteristics of excluded participants due to incomplete data were shown in Supplementary Table S1: they were older, had lower prevalence of hypertension (27.87 vs 30.55%), lower proportion of married status (78.46 vs 80.87%) and current smoker (28.63 vs 32.28%), and had slower gait speed (0.75 vs 0.78) than the included participants.

**TABLE 1:** Characteristics and comparing between groups.

	Participants without MCR (N = 5,311)	Participants with MCR (N = 414)	*p*-Value
Age, years (mean ± SD)	67.71 ± 6.71	68.40 ± 5.96	< 0.001
Male, n (%)	2,728 (51.37%)	138 (33.33%)	< 0.001
Urban residence, n (%)	1935 (36.46%)	105 (25.52%)	< 0.001
Married, n (%)	4,299 (80.95%)	330 (79.71%)	0.538
BMI, kg/m^2^ (mean ± SD)	23.37 ± 3.65	23.50 ± 3.89	0.06
Education, n (%)			< 0.001
No formal education or illiterate	2,843 (53.57%)	299 (72.22%)	
Primary or above	1,459 (27.49%)	81 (19.57%)	
Secondary or above	1,005 (18.94%)	34 (8.21%)	
Current Smokers, n (%)	1755 (33.04%)	93 (22.46%)	<0.001
Current Drinkers, n (%)	1,391 (26.19%)	71 (17.15%)	<0.001
History of hypertension, n (%)	1,543 (29.75%)	166 (40.69%)	<0.001
History of diabetes, n (%)	396 (7.69%)	40 (9.88%)	0.115
History of cardiac disease, n (%)	801 (15.46%)	80 (19.80%)	0.021
ADL disability, n (%)	1751 (32.97%)	273 (65.94%)	<0.001
Lower extremity functional limitation, n (%)	3,130 (58.93%)	345 (83.33%)	<0.001
Upper extremity functional limitation, n (%)	1,240 (23.35%)	219 (52.90%)	<0.001
Exhaustion, n (%)	1,057 (19.90%)	151 (36.47%)	<0.001
Inactivity, n (%)	501 (19.90%)	66 (28.33%)	<0.001
IPAQ score (Met/week)	54.75 ± 92.59	53.52 ± 89.77	0.002
Grip strength (mean ± SD, kg)	29.95 ± 12.11	24.44 ± 9.70	<0.001
2.5-m usual gait speed (mean ± SD, m/s)	0.81 ± 0.21	0.48 ± 0.11	<0.001
FTSS test time (s)	9.73 ± 4.10	10.99 ± 6.67	<0.001
Global cognitive function (mean ± SD)	10.58 ± 4.92	7.00 ± 4.25	<0.001

BMI, body mass index; ADL, activities of daily living; IPAQ, international physical activity questionnaire; FTSS, five times sit-to-stand.

### Associated Factors for MCR

Table 2 summarizes the results of multivariate logistic regression analyses. Model 1, adjusted for age, sex, living area, marital status, and education, shows that lower extremity functional limitations (OR: 2.96, 95% CI: 2.26–3.88), upper extremity functional limitations (OR: 3.16, 95% CI: 2.56–3.90), ADL disability (OR: 3.35, 95% CI: 2.70–4.17), grip strength (OR: 0.94, 95% CI: 0.92–0.95), FTSS test time (OR: 1.05, 95% CI: 1.02–1.07), exhaustion (OR: 2.07, 95% CI: 1.67–2.57), physical inactivity (OR: 1.51, 95% CI: 1.11–2.06), hypertension (OR: 1.62, 95% CI: 1.31–2.00), cardiac disease (OR: 1.44, 95% CI: 1.11–1.87), and diabetes (OR: 1.46, 95% CI: 1.03–2.07) are associated with MCR. In fully adjusted Model 2 in which all selected associated factors from Model 1 were entered together, lower extremity functional limitations (OR: 1.60, 95% CI: 1.02–2.50), upper extremity functional limitations (OR: 1.60, 95% CI: 1.11–2.30), ADL disability (OR: 1.86, 95% CI: 1.28–2.72), grip strength (OR: 0.96, 95% CI: 0.93–0.98), exhaustion (OR: 1.87, 95% CI: 1.33–2.63), and hypertension (OR: 1.48, 95% CI: 1.04–2.13) remained associated with MCR among the modifiable associated factors examined. In fully adjusted Model 2, none of the lifestyle factors examined were significantly associated with MCR.

**TABLE 2:** Associated factors for MCR.

	Model 1		Model 2	
Variables	Or (95%CI)	*p*	Or (95%CI)	*p*
Lifestyle Factors				
Current Smoking	0.95 (0.69–1.31)	0.750	0.96 (0.57–1.62)	0.887
Current Drinking	0.82 (0.61–1.11)	0.199	1.15 (0.70–1.89)	0.582
Overweight	1.36 (0.99–1.87)	0.061	0.99 (0.60–1.63)	0.977
Self-reported function limitation				
Lower extremity functional limitation	2.96 (2.26–3.88)	<0.001	1.60 (1.02–2.50)	0.043
Upper extremity functional limitation	3.16 (2.56–3.90)	<0.001	1.60 (1.11–2.30)	0.011
Disability				
ADL disability	3.35 (2.70–4.17)	<0.001	1.86 (1.28–2.72)	0.001
Physical performance				
Grip strength	0.94 (0.92–0.95)	<0.001	0.96 (0.93–0.98)	0.002
FTSS test time	1.05 (1.02–1.07)	<0.001	1.00 (0.96–1.03)	0.763
Component of frailty				
Exhaustion	2.07 (1.67–2.57)	<0.001	1.87 (1.33–2.63)	<0.001
Physical Activity				
Physical Inactivity	1.51 (1.11–2.06)	0.008	1.29 (0.86–1.92)	0.218
IPAQ score	1.00 (0.99–1.00)	0.651	1.00 (0.99–1.00)	0.636
Self-reported diseases				
Hypertension	1.62 (1.31–2.00)	<0.001	1.48 (1.04–2.13)	0.032
Cardiac disease	1.44 (1.11–1.87)	0.007	0.91 (0.57–1.39)	0.365
Diabetes	1.46 (1.03–2.07)	0.033	1.53 (0.89–2.12)	0.231

ADL, activities of daily living; FTSS, five times sit-to-stand; IPAQ, international physical activity questionnaire.

### Biomarker Correlates of MCR

We observed differences in systolic BP, diastolic BP, hemoglobin, cystatin C, CRP, and fasting glucose between participants with and without MCR (Table 3). When biomarkers were dichotomized using the lowest or the highest quintile (whichever indicated a harmful level) or clinically relevant cut-points, MCR individuals were more likely to have elevated levels of systolic BP, cystatin C, and CRP, and low levels of hematocrit, hemoglobin, and HDL cholesterol, than individuals without MCR. In fully adjusted model, higher CRP was significantly associated with MCR as categorical variable (OR: 1.45, 95% CI: 1.02–2.07, ≥2.8 mg/L vs < 2.8 mg/L); higher cystatin C was significantly associated with MCR both as categorical variable (OR: 1.53, 95% CI: 1.04–2.26, ≥1.0 mg/L vs < 1.0 mg/L) and continuous variable (OR: 3.95, 95% CI: 1.98–7.88, per 1.0 mg/L increase) (Table 4).

**TABLE 3:** Association of clinical measures with MCR.

	Participants without MCR (N = 5,311)	Participants with MCR (N = 414)	*p*-Value
SBP (mmHg)	132.04 ± 20.33	135.06 ± 23.65	0.006
SBP ≥150 mmHg,%	970 (18.26%)	110 (26.57%)	<0.001
DBP (mmHg)	75.8 ± 9.22	75.50 ± 10.19	0.006
DBP ≥90 mmHg,%	311 (5.86%)	32 (7.73%)	0.122
WBC count, 10^9^/L	5.97 ± 1.83	6.10 ± 1.82	0.487
WBC count ≥6.9 × 10^9^/L, %	1,313 (24.72%)	117 (28.26%)	0.109
Hematocrit, %	41.34 ± 5.51	40.36 ± 5.36	0.154
Hematocrit <38%, %	1,249 (23.52%)	122 (29.47%)	0.006
Platelets, 10^9^/L	199.03 ± 73.92	196.58 ± 69.85	0.626
Platelets ≥237 ×10^9^/L, %	1,371 (25.81%)	108 (26.09%)	0.903
Hemoglobin, g/dL	13.63 ± 1.87	13.27 ± 1.74	0.001
Hemoglobin <12 g/dl, %	792 (14.91%)	84 (20.29%)	0.003
Cystatin C, mg/L	0.92 ± 0.24	0.99 ± 0.50	<0.001
Cystatin C ≥ 1.0 mg/L	1,265 (23.82%)	132 (31.88%)	<0.001
CRP, mg/L	2.94 ± 6.27	3.58 ± 5.96	<0.001
CRP ≥2.8 mg/L,%	1,259 (23.71%)	124 (29.95%)	0.004
Fasting glucose, mg/dL	104.71 ± 34.68	109.83 ± 51.00	0.005
Fasting glucose≥125 mg/dl, %	662 (12.46%)	61 (14.73%)	0.181
LDL cholesterol, mg/dL	103.87 ± 29.01	101.04 ± 28.43	0.281
LDL cholesterol ≥121 mg/dl, %	1,351 (25.44%)	92 (22.22%)	0.147
HDL cholesterol, mg/dL	51.90 ± 12.14	50.64 ± 10.76	0.997
HDL cholesterol <43 mg/dl, %	1,277 (24.04%)	103 (24.88%)	0.048
Total cholesterol, mg/dL	185.34 ± 36.50	182.95 ± 35.00	0.931
Total cholesterol ≥207 mg/dl, %	1,343 (25.29%)	98 (23.67%)	0.466
Triglycerides, mg/dL, median	135.83 ± 83.90	144.56 ± 89.90	0.449
Triglycerides ≥162 mg/dl, %	1,331 (25.06%)	116 (28.02%)	0.182

SBP, systolic blood pressure; DBP, diastolic blood pressure; WBC, white blood cell; CRP, C-reactive protein; LDL, low-density lipoproteins; HDL, high-density lipoproteins.

**TABLE 4:** Logistic regression analyses of associations between clinical measures and MCR.

	Model 1	Model 2
Variables	OR (95%CI)	OR (95%CI)
CRP^a^	1.01 (1.00, 1.02)	1.01 (0.99, 1.03)
CRP^b^	1.37 (1.10, 1.71)	1.45 (1.02, 2.07)
Cystatin C^a^	1.93 (1.46, 2.57)	3.95 (1.98, 7.88)
Cystatin C^b^	1.50 (1.20, 1.86)	1.53 (1.04, 2.26)

OR, odds ratio; CI, confidence interval; CRP, C-reactive protein.

Baseline Model 1: tested each independent variable individually adjusted for demographic data (age, sex, living area, marital status, and education).

Fully adjusted Model 2: adjusted for age, gender, marital status, education level, living area, BMI, current smoking status, current drinking status, hypertension, diabetes, cardiac disease, grip strength, FTSS, test time; ADL, disability, lower extremity functional limitation, upper extremity functional limitation, exhaustion, inactivity and IPAQ, score.

a Continuous variable per one unit increase.

b Categorical variable (CRP, ≥ 2.8 mg/L vs < 2.8 mg/L; Cystatin C, ≥ 1.0 mg/L vs < 1.0 mg/L).

## Discussion

In this large, nationally representative sample of Chinese community-dwelling older adults, we found that 7.3% of Chinese adults aged 60 years or older have MCR syndrome. We found lower or upper extremity functional limitations, ADL disabilities, weaker grip strength, exhaustion, and history of hypertension to be significantly associated with higher odds of MCR syndrome. Further analysis of clinical measures showed that CRP and cystatin C were significantly correlated with MCR.

Our study estimated that the overall prevalence of MCR syndrome was 7.3% among Chinese community-dwelling older adults, which is lower than the prevalence found by The Ningbo Community Study on Aging (12.7%) (Zhang et al., 2020) and The Beijing Longitudinal Study of Ageing II (9.6%) (Chhetri et al., 2020). The differences might be due to the age distribution, size, and representativeness of samples, the definition of low gait speed, and the regional health disparities in China (Mu, 2014; Miao and Wu, 2016). Previous studies among Japanese elders (Nakamura et al., 2013; Doi et al., 2017) have revealed a higher prevalence of MCR among participants recruited from memory clinics (13%), and a lower prevalence of MCR among community-based respondents (6.4%). The source of the population sample may, therefore, impact the recorded prevalence of MCR. Another global epidemiological study based on responses from 22 cohorts from 17 countries reported a pooled prevalence of 9.7% with a variance of between 2 and 16% across cohorts (Verghese et al., 2014). Some single-center studies in Mexico (Aguilar-Navarro et al., 2019) and Malaysia (Lau et al., 2019) have reported the prevalence of MCR syndrome to be 14.3 and 3.4%, respectively. These differences in prevalence may potentially result from differences in the sociodemographic characteristics of the studied populations, and differences in ethnicity, behavior, and lifestyle.

We found that weakness is significantly associated with higher odds of MCR syndrome, which is in line with previous studies (Zhang et al., 2020). There are several potential pathophysiological mechanisms underlying the association between low handgrip strength and dementia, including production of pro-inflammatory cytokines (Hatabe et al., 2020), serum testosterone level, and white matter hyper-intensities in the brain (McGrath et al., 2020). Moreover, higher handgrip strength may be a proxy for engagement in habitual exercise, which has been found to be a protective factor for dementia (Lamb et al., 2018). We also found exhaustion and ADL disability to be significantly related to MCR syndrome, a result that has not been reported before. Similar to weakness, exhaustion is one of the indicators of frailty (Hatabe et al., 2020), which shares underlying pathogeneses with Alzheimer’s disease, including vascular pathology, dysregulated energy production, and stress (Islamoska et al., 2019). We found that FTSS time was only associated with MCR in the minimally adjusted model; when all potential confounders were adjusted for, this association was not statistically significant. This result is inconsistent with Zhang’s study (Zhang et al., 2020), which showed FTSS time to be significantly associated with MCR syndrome. However, both studies used a cross-sectional design, and this association needs to be further examined by prospective cohort studies in the future. In the present study, lifestyle factors, such as obesity, smoking status, and drinking status, were not associated with MCR after adjusting for all potential confounders. There have been numerous studies on the associations between lifestyle factors and dementia. However, few studies have focused on the associations between lifestyle factors and MCR syndrome. Two previous studies of the Chinese population showed that obesity and MCR syndrome are not correlated (Chhetri et al., 2020; Zhang et al., 2020), which is in line with our results. Notably, a recent meta-analysis indicated that a BMI ≥30 is not linked with an increased risk of all-cause dementia and non-vascular dementia (Lee et al., 2020). Consistent with the work of Chhetri and Zhang, we found no association between MCR and drinking status. However, while their work identified an association between MCR and smoking, we found no such association in the present study. Previous studies have confirmed that smoking, as a major cardiovascular risk factor, is associated with an increased risk of cognitive decline (Baumgart et al., 2015). It is important to note that there may be subtypes of MCR, specifically those that are characterized both by subjective cognitive complaints and objective cognitive decline, and those that have only subjective cognitive complaints without objective cognitive decline. The high proportion of MCR patients without objective cognitive decline might render the association between smoking and MCR insignificant. Moreover, cross-sectional study designs might not be robust enough to accurately capture these associations.

We observed an elevated level of CRP, an important marker of inflammation, among older adults with MCR. The associations between inflammatory markers, cognitive performance, (Baumgart et al., 2015; Chaparro and Suchdev, 2019; Qin et al., 2019), and gait speed (Verghese et al., 2011) have been repeatedly reported. Our study was the first of its kind to link elevated levels of CRP to MCR, which further supports the view that inflammation plays a crucial role in pathological declines in cognitive performance and gait speed. Additionally, we found MCR patients to have higher SBP levels than those without MCR, which agrees with the finding that participants with a history of hypertension have 1.48 times higher odds of having MCR syndrome. Similar findings were reported in a study by Zhang and others (Zhang et al., 2020). Furthermore, in the present study, we found that 20.29% of MCR patients had a hemoglobin concentration below 12 g/dl, the cutoff for anemia defined by the World Health Organization (Chaparro and Suchdev, 2019), as opposed to 14.91% among participants without MCR. This finding is consistent with evidence that anemia is an independent risk factor for cognitive decline (Qin et al., 2019) and frailty (Ruan et al., 2019). Notably, the multivariate analyses showed no statistically significant association between SBP, hemoglobin and MCR. Future studies are still warranted to further investigate these associations. Moreover, compared to the participants without MCR, proportionally more MCR patients had an elevated level of cystatin C (31.88 vs 23.82%, *p* < 0.001), a marker of kidney function (Jensen et al., 2017). The multivariate analysis also showed that cystatin C were significantly associated with MCR both as continuous and categorical variable. Previous studies have found that patients with chronic kidney disease (CKD) who are treated with dialysis have impaired physical functioning (Painter and Marcus, 2013), and impaired kidney function has been associated with impairments in learning and memory, language, complex attention, executive function, and global cognitive function among elders (Månsson et al., 2019).

Our study has several strengths. First, to our knowledge, this is the first study to examine the prevalence of MCR in a representative cohort of Chinese older adults. Second, our study offers comprehensive insight into the variety of factors associated with MCR syndrome by accounting for physical performance and clinical measures in community-dwelling Chinese older adults. Our findings will further the understanding of the pathophysiological mechanisms underlying MCR syndrome and the development of deep multifactorial interventions for MCR syndrome. However, some limitations of our study should also be noted. Firstly, this analysis is based on cross-sectional data; therefore, we were unable to establish a causal association. Future studies utilizing a prospective design may elucidate a potential dynamic association between these related factors. Second, selection bias may exist in this study since only community dwellers were enrolled in the baseline survey of the CHARLS. Therefore, our estimates of the prevalence of MCR may not generalize to the entirety of the Chinese elderly population. Thirdly, some modifiable associated factors were measured based on self-reported answers, such as ADL disabilities, functional limitations and cognitive function. This might be subject to recall bias and lead to overestimation or underestimation of exposure. Finally, traits associated with MCR were only measured once; these measures may vary over time. Therefore, future research must characterize the trajectories of MCR over time, and further explore their relation to adverse outcomes, including dementia, falls, and mortality.

## Conclusion

Our large cohort study reported that 7.3% of Chinese adults aged 60 years or older have MCR syndrome. Additionally, lower or upper extremity functional limitations, ADL disabilities, weaker grip strength, exhaustion, and history of hypertension were observed to be significantly associated with higher odds of MCR syndrome. Our results also showed that clinical measures including cystatin C and CRP were correlated with MCR. Our findings will help improve early detection and develop prevention strategies for dementia in China and other countries, and more longitudinal studies are warranted to further explore the modifiable risk factors of MCR.

## Data Availability

The original contributions presented in the study are included in the article/Supplementary Materials, further inquiries can be directed to the corresponding author.
