# Supplementary material for: Prevalence and Correlates of Motoric Cognitive Risk Syndrome in Chinese Community-Dwelling Older Adults
Source: Front Aging. 2022 Jun 30;3:895138. doi: 10.3389/fragi.2022.895138 (PMC9261413; doi:10.3389/fragi.2022.895138)
Supplement: Supplementary file 1 [file Table1.DOCX]

Table S1. Sample Characteristics and comparing between Complete Data and Incomplete Data

|  | Total Sample (N=7517) | Complete Data  (N=5725) | Incomplete Data (N=1792) | P-value |
| --- | --- | --- | --- | --- |
| Age, years (mean ± SD) | 68.01±6.70 | 67.88 ± 6.63 | 68.44 ± 6.88 | <0.001 |
| Male, n(%) | 3737 (49.71 %) | 2866 (50.06%) | 871 (48.60%) | 0.282 |
| Urban residence, n (%) | 2458 (35.59%) | 2040 (35.66%) | 418 (35.21%) (N=1187) | 0.768 |
| Married, n (%) | 6035 (80.28%) | 4418 (80.87%) | 1406 (78.46%) | 0.026 |
| BMI, kg/m^2^ (mean ± SD) | 23.44 ± 4.14 | 23.38 ± 3.67 | 23.62 ± 5.41 (N=1757) | 0.274 |
| Education, n (%) |  |  |  | 0.991 |
| No formal education or illiterate | 4033 (54.96%) | 3142 (54.92%) | 891 (55.10%) (N=1617) |  |
| Primary or above | 1973 (26.89%) | 1540 (26.92%) | 433 (26.78%) |  |
| Secondary or above | 1332 (18.15%) | 1039 (18.16%) | 293 (18.12%) |  |
| Current Smokers, n (%) | 2361 (31.41%) | 1848 (32.28%) | 513 (28.63%) | 0.012 |
| Current Drinkers, n (%) | 1891 (25.16%) | 1462 (25.54%) | 429 (23.94%) | 0.341 |
| History of hypertension, n (%) | 2124 (29.99%) | 1709 (30.55%) | 415 (27.87%) (N=1489) | 0.045 |
| History of diabetes, n (%) | 540 (7.68%) | 436 (7.85%) | 104 (7.06%) (N=1474) | 0.309 |
| History of Coronary Heart Disease, n (%) | 1114 (15.75%) | 881 (15.78%) | 233 (15.65%) (N=1489) | 0.903 |
| ADL disability, n(%) | 2652 (35.28%) | 2024 (35.35%) | 628 (35.04%) | 0.811 |
| Lower extremity functional limitation, n (%) | 4556 (60.61%) | 3475 (60.70%) | 1081 (60.32%) | 0.777 |
| Upper extremity functional limitation, n (%) | 1923 (25.58%) | 1459 (25.48%) | 464 (25.89%) | 0.730 |
| Exhaustion, n(%) | 1604 (21.34%) | 1208 (21.10%) | 396 (22.10%) | 0.368 |
| Inactivity, n(%) | 741 (20.41%) | 567 (20.61%） | 174 (19.80%) (N=879) | 0.602 |
| IPAQ score (Met/week) | 53.39 ± 93.55 | 54.67 ± 92.39 | 57.70 ± 97.14 | 0.668 |
| Grip strength, (mean ± SD, kg) | 29.14 ± 9.17 | 29.33 ± 9.09 | 28.52 ± 9.40 (N=1398) | 0.820 |
| 2.5 meter usual gait speed (mean ± SD, m/s) | 0.77 ± 0.25 | 0.78 ± 0.22 | 0.75 ± 0.31 | <0.001 |
| FTSS test time(s) (mean ± SD) | 9.88 ± 4.55 | 9.82 ± 4.34 | 10.06 ± 5.15 | 0.243 |
| Global cognitive function (mean ± SD) | 10.31 ± 4.97 | 10.32 ± 4.99 | 10.26 ± 4.94 | 0.153 |

BMI, body mass index; ADL, activities of daily living; IPAQ, international physical activity questionnaire; FTSS, five times sit-to-stand.
